# Serum paraoxonase-1 as a marker of oxidative stress and pulmonary dysfunction in sarcoidosis: association with disease activity and prognostic potential

**DOI:** 10.3389/fimmu.2025.1731991

**Published:** 2026-01-12

**Authors:** Mine Büşra Bozkürk, Ersin Günay, Nalan Ogan, İpek Candemir, Uğurcan Eker, Alpaslan Öztürk

**Affiliations:** 1 Department of Clinical Biochemistry, Etlik City Hospital, Ankara, Türkiye; 2 Department of Chest Diseases, Etlik City Hospital, Ankara, Türkiye

**Keywords:** antioxidant enzyme, biomarker, oxidative stress, paraoxonase-1, pulmonary function tests, sarcoidosis

## Abstract

**Objectives:**

Sarcoidosis is a multisystem granulomatous disorder characterized by an oxidative imbalance and inflammatory activation. Paraoxonase-1 (PON1) is a high-density lipoprotein–associated antioxidant enzyme that plays a protective role against oxidative stress. This study aimed to evaluate the serum PON1 activity in patients with sarcoidosis, its relationship with disease stage, and its association with pulmonary function and routine biochemical parameters.

**Materials and methods:**

A total of 45 patients with sarcoidosis and 45 age- and sex-matched healthy controls were included. Complete blood counts, biochemical parameters, and pulmonary function test results were evaluated. Serum PON1 activity was colorimetrically determined using a commercial kit. Statistical analyses included correlation, logistic regression, receiver operating characteristic (ROC) curves, and partial least squares discriminant analysis (PLS-DA).

**Results:**

Serum PON1 activity was significantly lower in patients with sarcoidosis than in the healthy controls (p<0.001). PON1 levels showed strong positive correlations with FEV_1_ (r=0.835), FVC (r=0.807), and DLCO (r=0.905), indicating that decreased antioxidant capacity parallels impaired pulmonary function. ROC analysis demonstrated that PON1 effectively differentiated patients from controls (AUC = 0.838; sensitivity, 75%; specificity, 82% at ≤210.53 U/L). PON1 levels declined progressively with increasing disease stages (p<0.05). Logistic regression analysis revealed that higher BMI and reduced FEV_1_ were independent predictors of sarcoidosis, whereas PON1 lost significance after adjusting for covariates.

**Conclusion:**

Serum paraoxonase activity is markedly reduced in sarcoidosis and is correlated with disease severity and pulmonary dysfunction, suggesting increased oxidative stress. Although not an independent predictor, PON1 may serve as a useful adjunctive biomarker, particularly for evaluating disease severity in Stage II and advanced stages, rather than for early diagnosis.

## 1 Introduction

Sarcoidosis is a chronic disease characterized by systemic non-caseating granulomas, the etiology of which is not yet fully understood. Genetic and environmental factors are thought to play a role in this pathophysiology. Approximately 90% of cases affect the lungs and intrathoracic lymph nodes. Peripheral lymph nodes, skin, eyes, liver, spleen, muscles, bones, upper respiratory tract, heart, nerves, urinary system, and many other organs may also be involved in 10-30% of patients (1, 2).

Specific and sensitive biomarkers for sarcoidosis can reduce the number of radiological examinations required for disease management. Therefore, various biomarkers have been investigated to determine the diagnosis, activity, and progression of this disease (3). However, despite recent advances in understanding the mechanisms involved in the development of sarcoidosis, the difficulty in diagnosing sarcoidosis persists, and there are still no validated biomarkers that can assist clinicians in the diagnosis or prediction of outcomes (4).

Oxidative stress is defined as an imbalance between the production and excretion of oxidant compounds and may underlie some of the molecular mechanisms that characterize the pathogenesis of sarcoidosis. The role of oxidative stress has been extensively documented in various lung diseases, including chronic obstructive pulmonary disease (COPD) and lung fibrosis (5, 6). Although studies on oxidative stress in sarcoidosis have been less frequent in recent years than those on other diseases, the presence of redox imbalance in sarcoidosis has also been demonstrated (7).

Serum paraoxonase-1 (PON1) is a glycoprotein that binds to circulating high-density lipoprotein (HDL) particles and protects their function by inactivating lipid peroxidation. It has also been shown to inhibit low-density lipoprotein (LDL) oxidation, thereby protecting endothelial cells from apoptosis (8). The presence of PON1 in type 1 pneumocytes in the lung has been confirmed by immunohistochemical methods; however, its physiological role in the context of lung antioxidant activity has not yet been defined (9). However, there are few reports on the role of PON1 activity in patients with asthma and COPD (10–12), and there are reports on its effect on lung cancer behavior and progression through antioxidant function and controlled ROS accumulation (13).

An association between PON-1 activity and many diseases has been reported. These conditions include atherosclerosis (14), systemic lupus erythematosus (15), malignant tumors (16), Alzheimer’s disease (17), psoriasis (18), chronic renal failure (19), and asthma (20). While there are some studies on sarcoidosis and its oxidative status (21, 22), no studies have reported the relationship between PON-1 levels and the stage of this disease.

Due to the relationships and potential correlations described above, our study aimed to investigate the relationship between serum paraoxonase levels in sarcoidosis patients and disease stage and laboratory parameter results (complete blood count and routine biochemistry tests).

## 2 Material and methods

### 2.1 Study setting and study population

The study protocol was approved by the Ankara Etlik City Hospital Ethics Committee (dated: 02.07.2025; acceptance number: AEŞH-BADEK-1-2025-051). Signed consent forms were obtained from all individuals included in the study.

A total of 45 patients aged 18–80 who were diagnosed with sarcoidosis at the * Hospital Chest Diseases outpatient clinic were included in this study. To compare these patient groups, a healthy control group of 45 patients was included in this study. The healthy control group consisted of individuals without chronic diseases and no regular medication use. The healthy control group consisted of individuals with no history of chronic or acute inflammatory disease, malignant neoplasia, alcoholism, or any other systemic condition. All controls were clinically evaluated to confirm the absence of active disease. Patient groups were matched for age and sex after identification. Individuals diagnosed with alcoholism, chronic inflammatory disease, or history of malignant neoplasia were excluded from the study. These exclusion criteria were applied to both groups to ensure that the control group represented a healthy population without systemic diseases. Complete blood counts and routine biochemistry tests were performed on the same day from venous blood samples obtained from the patients participating in our study and healthy controls. The remaining samples from routine biochemistry tests were aliquoted and stored at -80 °C until the study day. Serum PON-1 activity was measured once a day by colorimetric testing using commercially available kits (Relassay, Turkey). The rate of paraoxon hydrolysis (diethylnitrophenylphosphate) was measured by monitoring the increase in absorption at 412 nm at 37 °C. The amount of p-nitrophenol generated was calculated from the molar absorption coefficient at pH 8.5, and was 18.290 M−1 cm−1. Paraoxonase activity is expressed in U/L. In this study, PON1 activity was assessed using a colorimetric paraoxon hydrolysis assay. Arylesterase activity, which reflects the total catalytic capacity of PON1, was not measured; therefore, our analysis was limited to paraoxonase-specific activity rather than overall PON1 functionality. All PON1 measurements were performed following strict quality control procedures. Assays were conducted using fresh substrate solutions, and calibration curves were generated for each batch of the enzyme. Internal controls were run in duplicate for each plate to monitor intra-assay variability, and samples were analyzed in random order to minimize batch effects. The coefficient of variation (CV) for repeated measurements remained within the acceptable range (<10%), indicating stable assay performance and reliable quantification of the analytes. Patient demographic data (age, sex, body mass index (BMI), complete blood count parameters, routine biochemistry tests (urea, creatinine, glucose, alanine aminotransferase (ALT), aspartate aminotransferase (AST)), sarcoidosis disease stage) and clinical data were recorded.

### 2.2 Statistical analysis

All analyses were performed using IBM SPSS Statistics v23 (IBM Corp., Armonk, NY), MedCalc v23.3.7 (MedCalc Software, Ostend, Belgium), and MetaboAnalyst (www.metaboanalyst.ca; web-based platform). Distributions of continuous variables were assessed using the Shapiro–Wilk test and Q–Q plots, and homogeneity of variance was assessed using the Levene test. Normally distributed variables were summarized using the mean ± SD, non-normally distributed variables were summarized using the median (p25–p75), and categorical variables were summarized using the number (%). For comparisons of the two groups (sarcoidosis *vs*. healthy individuals), independent samples t-tests or Mann–Whitney U tests were used depending on the distribution; for categorical variables, the chi-square test (Fisher’s exact test, if necessary) was used. For comparisons between the four stages, one-way ANOVA (Tukey HSD *post hoc*) or Kruskal–Wallis test (Dunn–Bonferroni *post hoc*) was applied, depending on the distribution.

The effect sizes were reported as Cohen’s d for parametric comparisons, ordinal effect size r (z/√N) for nonparametric comparisons, and Cramér’s V for categorical variables. The two-tailed significance level was set at α=0.05; additional multiple comparison correction was performed only for *post-hoc* tests between stages; and other hypothesis tests were interpreted as exploratory.

Relationships between variables were examined using Spearman’s rank correlation; correlation matrices and scatter plots were plotted, and linear trend lines were added to visualize trends.

Univariate and multivariate logistic regression models were constructed to evaluate predictors associated with sarcoidosis. Candidate covariates included age, sex, BMI, NLR, FEV_1_, FVC, and paraoxonase (PON1) activity based on clinical significance and univariate analysis results. All covariates were included in the model using the enter method. Multicollinearity was assessed using the variance enhancement factor (VIF) (VIF >10 was considered an indicator of significant collinearity). Model fit was assessed using the Hosmer–Lemeshow test and classification statistics; odds ratios (OR) and 95% confidence intervals (CIs) were reported.

Receiver operating characteristic curve analysis was performed using MedCalc to assess the discriminatory power of PON1. The area under the curve (AUC) and 95% confidence intervals (CIs) were calculated using the DeLong method. The optimal threshold value was determined using the Youden index (J = sensitivity + specificity − 1), and 95% confidence intervals for sensitivity and specificity were calculated.

Partial least squares discriminant analysis (PLS-DA) was conducted using MetaboAnalyst to examine the multivariate patterns and to handle multicollinearity between respiratory function parameters. The model input variables included FEV1, FVC, BMI, NLR, and Age. Before modeling, continuous variables were autoscaled to have a mean of zero and a variance of one. Score plots and group ellipses were generated to assess model discrimination, and VIP scores and biplots were generated to assess variable contributions. To reduce overfitting, the platform’s cross-validation and permutation diagnostic outputs were observed and interpreted (R²/Q² trends were inspected qualitatively).

A complete-case approach was adopted for the variables with no missing data. Outliers were assessed graphically, and the analysis was repeated only when there was a clear indication of data error. All tests were two-tailed, and statistical significance was set at p<0.05.

## 3 Results

### 3.1 Baseline characteristics of the study population

This study included 45 patients with sarcoidosis and 45 healthy individuals as controls. The basic demographic and anthropometric data of the study groups are summarized in **Table 1**. No statistically significant difference was found in terms of sex distribution between the sarcoidosis patient group (25 females, 20 males) and the healthy control group (24 females, 21 males). Similarly, no significant differences were observed in the mean age of the groups. These data indicate that the study groups were successfully matched with respect to basic demographic variables such as age and sex, and that the effect of these potential confounding factors on the analysis results was minimized. In the comparison of anthropometric measurements, the median height of the control group was significantly higher than that of the patient group. However, no statistically significant differences were detected in terms of BMI and weight. The Hematological profile and systemic inflammation markers of the sarcoidosis patient group indicated the presence of a significant systemic inflammatory response compared to healthy controls (**Table 1**). The mean white blood cell (WBC) count was found to be statistically significantly higher in the patient group than in the control group. When the underlying causes of leukocytosis were examined, both the mean neutrophil count and median lymphocyte count were significantly higher in the patient group. No statistically significant difference was found between the groups in terms of the Neutrophil/Lymphocyte Ratio (NLR). Among other hematological findings, a slight but statistically significant decrease in the mean red blood cell (RBC) count was found in the patient group compared to that in the control group. The platelet count was significantly higher in patients with sarcoidosis than in controls. Observed Deterioration in Respiratory Functions The pulmonary effects of sarcoidosis are manifested by marked deterioration in pulmonary function tests (PFTs) (**Table 1**). In the patient group, the median forced expiratory volume in one second (FEV1) and median forced vital capacity (FVC) were found to be statistically significantly lower than the median values ​​in the healthy control group. A similar significant decrease was observed when these parameters were expressed as a percentage of the expected values. These decreases in lung volume suggest a restrictive pattern due to interstitial fibrosis and parenchymal involvement caused by sarcoidosis. Furthermore, the significantly lower FEV1/FVC ratio in the patient group than in the control group suggests that airway obstruction is also associated with the pathology. This finding suggests a mixed-type ventilation disorder, indicating the presence of endobronchial granulomas and reflecting the complex pathophysiology of the disease. When the diffusing capacity for carbon monoxide (DLCO), which assesses gas exchange function, was examined, the median DLCO value in the patient group was found to be significantly impaired compared to that in the control group. No significant differences were found between the groups in terms of glucose, urea, ALT, or AST levels. However, creatinine concentration was significantly higher in the patient group.

**Table 1:** Comparative analysis of demographic, anthropometric, hematological, biochemical, and respiratory function parameters in the sarcoidosis patient group and the healthy control group.

		Healthy control group (N = 45)	Sarcoidosis patient group (N = 45)	Effect size	Direction	P Value
Gender	Female	24	25			0.654
	Male	21	20			
Stage	Stage I	0	16			N.D.
	Stage II	0	12			
	Stage III	0	9			
	Stage IV	0	8			
Age (year)		48.16 ± 9.58	51.4 ± 12.8	0.284	↑ in Patients	0.123
Height		170 (164-179)	166 (158-176)	-0.211	↑ in Controls	0.045
Weight		73 (67-83)	80 (71-85)	0.156	↑ in Patients	0.140
BMI (kg/m^2^)		25.6 (25.1-26.1)	26.8 (25.7-30.5)	0.347	↑ in Patients	0.101
WBC (10^3^/μL)		7.3 ± 1.8	9.9 ± 1.6	1.514	↑ in Patients	0.001
RBC (10^6^/μL)		4.9 (4.4-5.5)	4.58 (4.5-4.64)	-0.212	↑ in Controls	0.044
Neutrophil (10^3^/μL)		4.7 ± 1.7	6.1 ± 1.9	0.77	↑ in Patients	0.001
Lymphocyte (10^3^/μL)		1.8 (1.3-2.4)	2.5 (2.3-2.7)	0.347	↑ in Patients	0.001
Platelet (10^3^/μL)		275 (200-348)	329 (302-380)	0.347	↑ in Patients	0.001
NLR		2.54 (1.78-3.75)	2.46 (2.04-2.83)	-0.107	↑ in Controls	0.309
Glucose (mg/dL)		88 (80-94)	88.1 (81.7-95.4)	0.048	↑ in Patients	0.651
Urea (mg/dL)		32.2 ± 8.47	30.2 ± 5.27	-0.281	↑ in Controls	0.508
Creatinine (mg/dL)		0.9 (0.8-1)	0.94 (0.85-1.01)	0.275	↑ in Patients	0.009
ALT (U/L)		24 (15-35)	24.1 (21.7-32.5)	0.131	↑ in Patients	0.215
AST (U/L)		26 (19-38)	22.8 (19.4-27.5)	-0.102	↑ in Controls	0.333
FEV1		3.3 (3-3.7)	2.2 (1.1-2.7)	-0.347	↑ in Controls	0.001
FEV1%		89 (85-92)	74.3 (63.6-83.8)	-0.347	↑ in Controls	0.001
FVC		3.8 (3.4-4.2)	2.99 (1.6-3.33)	-0.347	↑ in Controls	0.001
FVC %		89 (85-92)	79.9 (73.8-90.3)	-0.347	↑ in Controls	0.001
FEV1/FVC		88.2 (85.3-93.6)	76.9 (70.3-81.8)	-0.347	↑ in Controls	0.001
DLCO		93 (87-101)	71.1 (64.5-79.4)	-0.347	↑ in Controls	0.001
Paraoxonase (U/L)		275 (225-372)	110 (71.8-210)	-1.547	↑ in Controls	0.001

ALT, Alanine aminotransferase; AST, Aspartate aminotransferase; BMI, Body mass index; DLCO, Diffusing capacity of the lungs for carbon monoxide; FEV1, Forced expiratory volume in one second; FVC, Forced vital capacity; NLR, Neutrophil-to-lymphocyte ratio; Paraoxonase, Paraoxonase enzyme activity; RBC, Red blood cell; WBC, White blood cell.

### 3.2 Paraoxonase activity in sarcoidosis patients and controls

**Figure 1:**
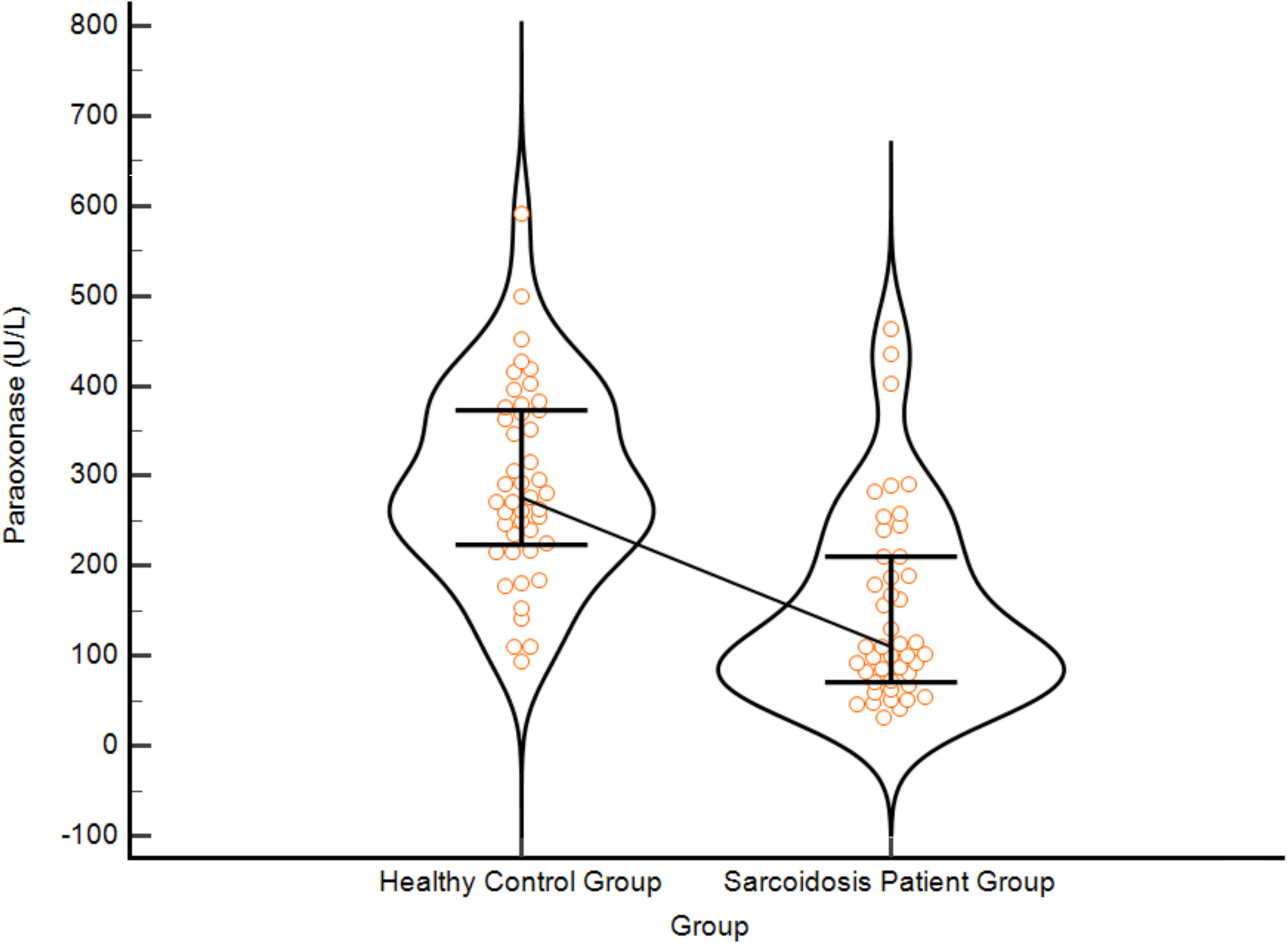
Comparison of serum paraoxonase (PON1) activity levels between the healthy control group and the sarcoidosis patient group. The violin plots display the distribution density of the data, with individual data points overlaid to show variability. The central bold horizontal lines represent the median values, while the upper and lower thinner lines indicate the interquartile range (25th–75th percentiles). Serum PON1 activity was found to be statistically significantly lower in sarcoidosis patients compared to healthy controls (p<0.001), indicating reduced antioxidant capacity in the disease state.

**Figure 2:**
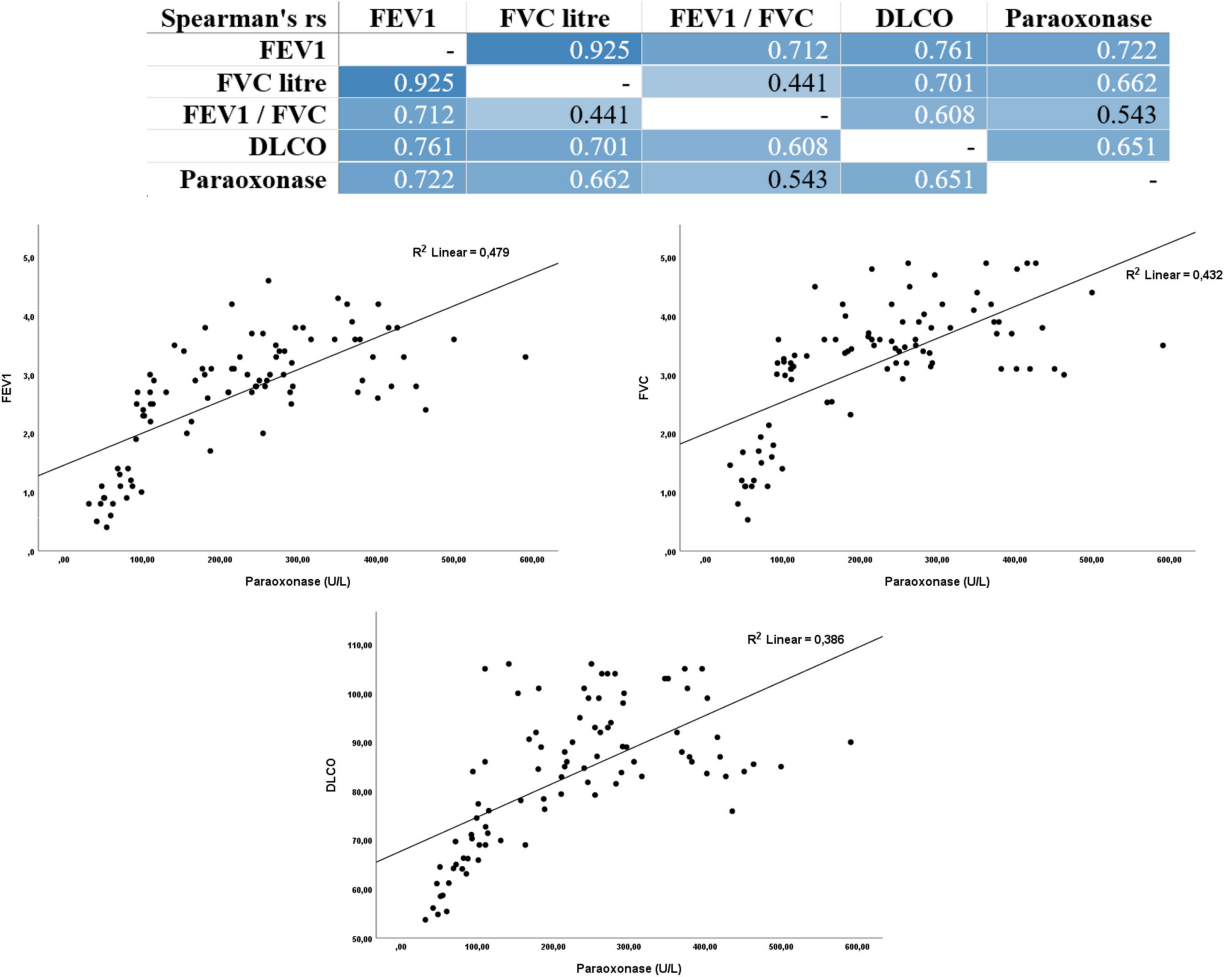
Correlations between serum paraoxonase activity and respiratory function parameters. The upper panel presents the Spearman’s rank correlation matrix, where darker blue shades indicate stronger positive correlations among FEV1, FVC, DLCO, and Paraoxonase. The lower panels show scatter plots with linear regression trend lines illustrating the positive relationship between Paraoxonase levels and FEV1 (R^2^ = 0.479), FVC (R^2^ = 0.432), and DLCO (R^2^ = 0.386). A decrease in PON1 activity is strongly associated with impaired pulmonary volumes and gas exchange capacity.

Serum PON-1 levels in the sarcoidosis patient group were statistically significantly lower than in the healthy control group (**Figure 1**). Serum paraoxonase activity correlated in the same direction as all major components of pulmonary function tests (**Figure 2**). Relationships between both forced expiratory volume and forced vital capacity were significant, and a high degree of coordination between these two volume-based measures was confirmed by the correlation matrix. The diffusion measure, which reflects gas exchange capacity, also demonstrated a significant and clinically significant positive relationship with paraoxonase. The correlation between the volume-based and rate-based measures, although more modest than the others, was consistently positive. Data points in the scatter plots indicate a steady increase in respiratory function parameters as paraoxonase activity increased. The linear trend lines present a pattern suggesting that these relationships are not only statistically significant but also practically meaningful, suggesting that the prediction line rises with a slope amenable to clinical interpretation, and that the relationships are not determined by individual outliers. Taken together, these findings support the idea that decreased antioxidant capacity (low paraoxonase activity) parallels the deterioration of respiratory function, and that the pathophysiological axis associated with oxidative stress may play an important role in shaping the pulmonary effects of the disease. While the detailed correlation coefficients and significance levels for all study parameters are provided in **Supplementary Table S1**, the specific interrelationships within the sarcoidosis patient group are visualized in the heatmap presented in **Supplementary Figure S1**. In this visualization, the color intensity corresponds to the magnitude of the Spearman’s rho coefficient, highlighting the strong clustering of respiratory and inflammatory parameters.

### 3.3 Correlation between PON1 activity and lung function parameters

In the univariate analyses, an increase in body mass index was positively associated with the likelihood of sarcoidosis. Regarding respiratory function, both forced expiratory volume in one second (FEV_1_) and forced vital capacity (FVC) showed strong inverse associations with sarcoidosis. Serum paraoxonase activity showed a protective association, with higher activity associated with a lower likelihood of sarcoidosis. A trend-level association was observed for the neutrophil-to-lymphocyte ratio, whereas age and sex were not significantly associated (**Table 2**).

**Table 2:** Predictors associated with the likelihood of sarcoidosis: Univariate and multivariate logistic regression analysis.

Univariate logistic regression						
	B	Wald	P value	Odds ratio (Exp(B))	95% CI for odds ratio	
					Lower	Upper
Gender	-0.09	0.045	0.832	0.914	0.399	2.097
Age (year)	0.024	1.554	0.213	1.024	0.986	1.063
BMI (kg/m2)	0.488	8.574	0.003	1.628	1.175	2.257
NLR	-0.393	3.632	0.057	0.675	0.451	1.011
FEV1	-4.737	16.362	0.001	0.0087	0.001	0.0097
FVC	-3.067	15.676	0.001	0.047	0.01	0.213
Paraoxonase (U/L)	-0.012	21.21	0.001	0.988	0.983	0.993
Multivariate logistic regression						
	B	Wald	P value	Odds ratio (Exp(B))	95% CI for odds ratio	
					Lower	Upper
Gender	-0.234	0.066	0.797	0.791	0.133	4.707
Age (year)	-0.032	0.395	0.530	0.969	0.877	1.070
BMI (kg/m2)	0.926	5.114	0.024	2.523	1.131	5.629
NLR	-0.691	1.540	0.215	0.501	0.168	1.492
FEV1	-5.380	6.457	0.011	0.005	0.001	0.292
FVC litre	-0.446	0.083	0.773	0.640	0.031	13.216
Paraoxonase (U/L)	-0.003	0.671	0.413	0.997	0.989	1.004

BMI, Body mass index; CI, Confidence interval; Exp(B), Exponentiated coefficient representing odds ratio; FEV1, Forced expiratory volume in one second; FVC, Forced vital capacity; NLR, Neutrophil-to-lymphocyte ratio; Paraoxonase, Paraoxonase enzyme activity.

Taken together, these findings indicate that a higher body mass index and impaired lung function were independently associated with the presence of sarcoidosis, whereas paraoxonase activity provided a protective signal only in unadjusted analyses and did not remain an independent predictor.

### 3.4 Diagnostic performance of PON1

**Figure 3:**
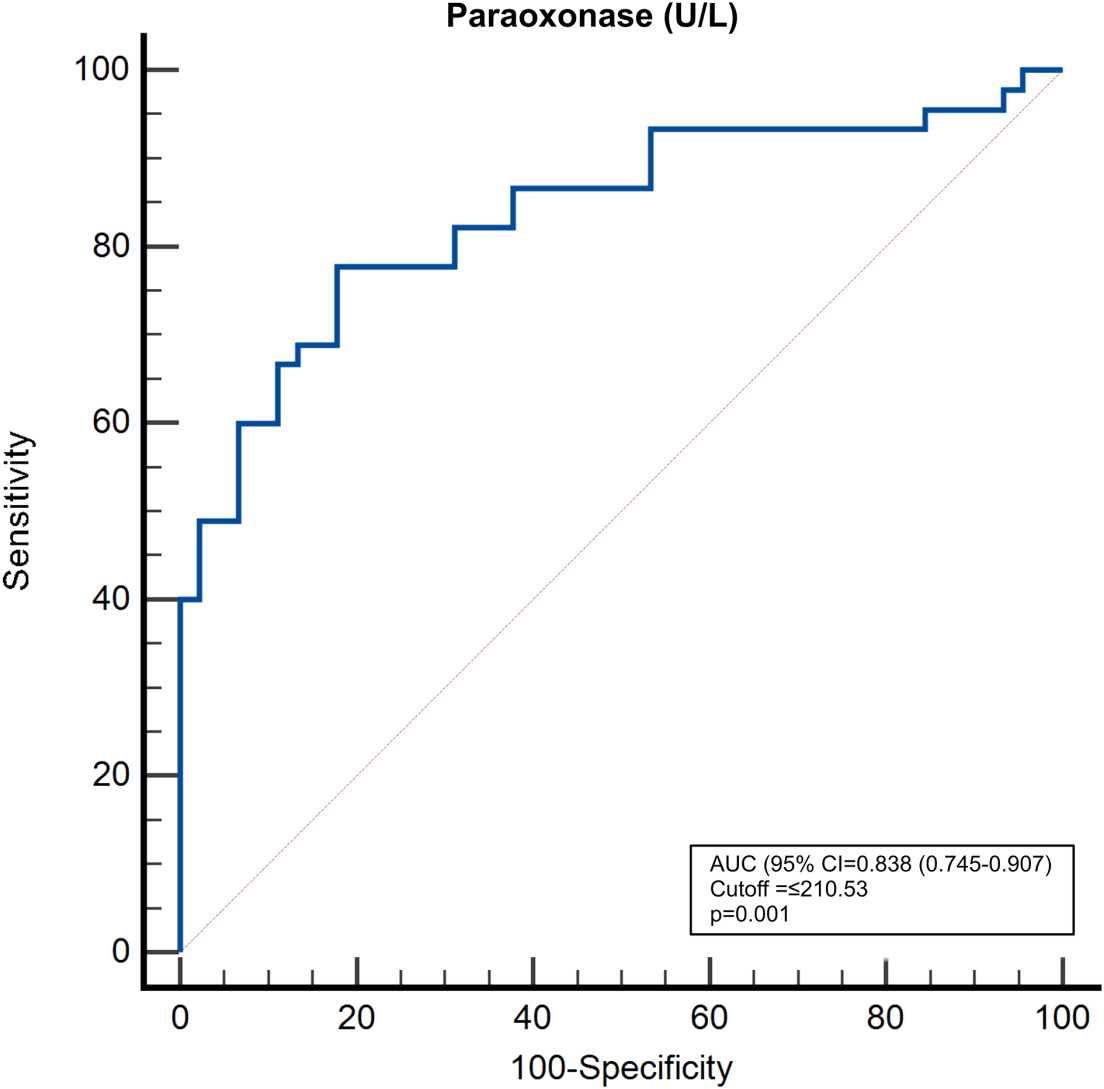
Receiver Operating Characteristic (ROC) curve analysis evaluating the diagnostic performance of serum paraoxonase activity in distinguishing sarcoidosis patients from healthy controls. The analysis yielded an Area Under the Curve (AUC) of 0.838 (95% CI: 0.745–0.907; p<0.001). A calculated cutoff value of ≤210.53 U/L demonstrated a sensitivity of 75% and a specificity of 82%, suggesting that PON1 is a discriminative biomarker for sarcoidosis.

Serum paraoxonase activity demonstrated significant accuracy in distinguishing sarcoidosis patients from healthy subjects (AUC = 0.838, 95% CI:0.745–0.907; p<0.001; **Figure 3**). At a threshold value of ≤210.53 U/L derived from the ROC curve, the sensitivity of the test was 75% (95% CI:60–87), and the specificity was 82% (95% CI:68–92). This pattern suggests that paraoxonase activity, even when applied alone, offers clinically significant discriminatory power and can indicate the likelihood of sarcoidosis with moderate-to-high accuracy. Measurements above the threshold value indicated a healthy subject, whereas those below the threshold value indicated a more likely presence of sarcoidosis; thus, paraoxonase emerged as a supportive biomarker in the preliminary diagnosis process.

### 3.5 PLS-DA findings (multivariate classification model)

In the multivariable model, all candidate variables were considered simultaneously, body mass index remained positively and independently associated with sarcoidosis. Among the respiratory indices, FEV_1_ retained an inverse, independent association after adjustment for covariates, whereas FVC did not remain significant, consistent with high collinearity among volume-based measures. The protective association of paraoxonase activity observed in the univariate analysis was attenuated and did not persist once lung function and body mass index were included, suggesting shared variance or mediating/overlapping mechanisms between paraoxonase and respiratory performance. The neutrophil-to-lymphocyte ratio, age, and sex were not independently associated with the likelihood of sarcoidosis in the multivariable analysis (**Table 2**).

**Figure 4:**
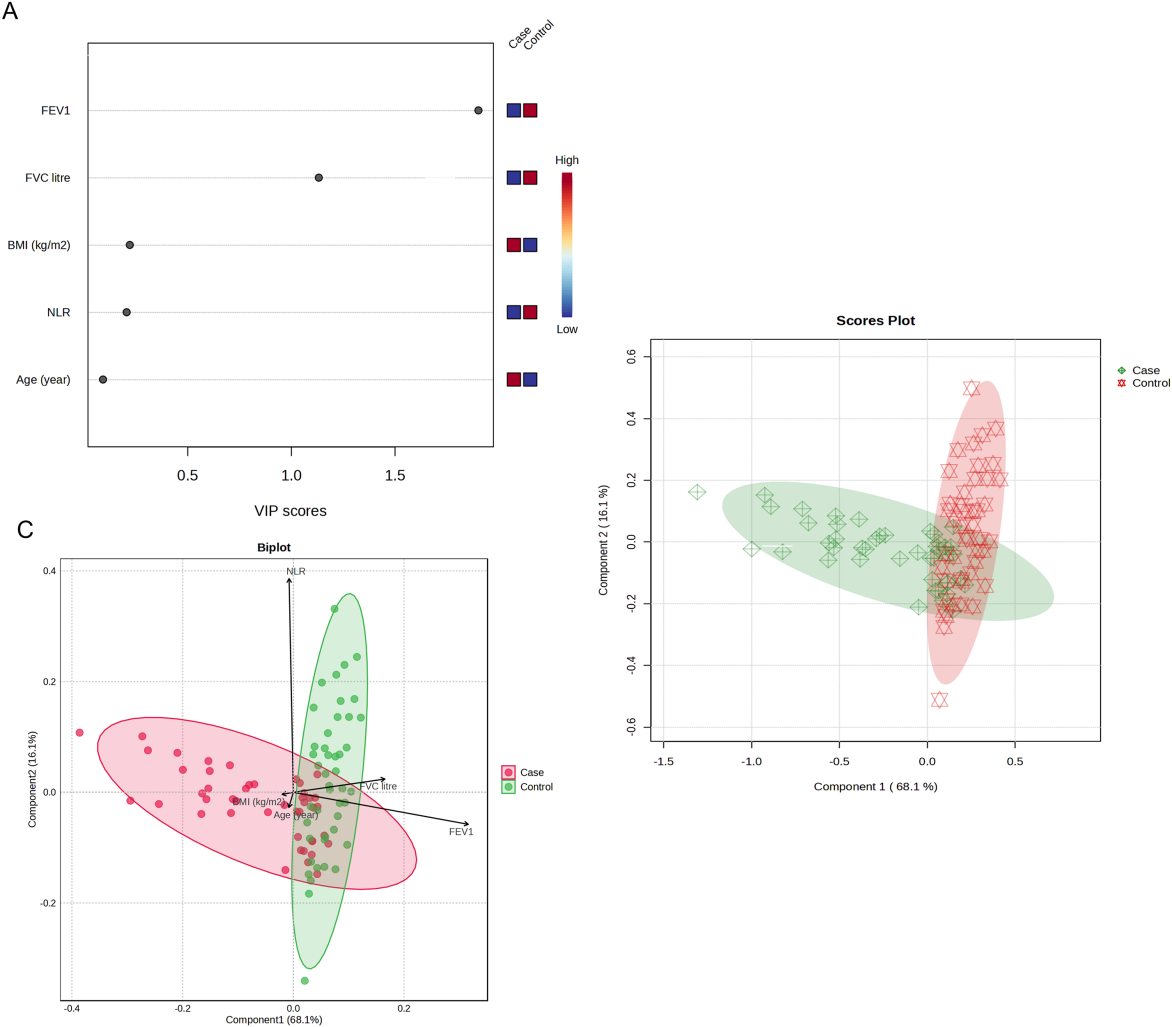
Partial Least Squares Discriminant Analysis (PLS-DA) results for the classification of sarcoidosis patients versus controls. **(A)** Variable Importance in Projection (VIP) scores identifying the most discriminatory variables; FEV1 and FVC contributed most to the model. **(B)** Biplot displaying the superposition of scores and loadings, visualizing the correlation structure. **(C)** 2D Score plot showing distinct clustering and separation between the Sarcoidosis group (red circles) and Healthy Control group (green diamonds) with 95% confidence ellipses.

By jointly evaluating respiratory function, inflammation, and demographic axes, the PLS-DA model revealed a clear distinction between cases and controls. The score plot (**Figure 4C**) showed that the distinction occurred primarily in the first latent component, with the second component carrying additional but more limited information, and the structure of the distribution suggested a consistent group pattern rather than isolated outliers. VIP scores (**Figure 4A**) revealed that the most discriminating variables were respiratory function measures; FEV_1_ ranked first, followed by FVC. BMI made a moderate contribution, whereas the relative contributions of NLR and age were more limited. The biplot (**Figure 4B**) showed that the FEV_1_ and FVC vectors were collinear with the discrimination axis and exhibited co-trending variation, with BMI making more moderate contributions to this axis, whereas NLR and age made weaker and partially orthogonal contributions. Taken together, these findings suggest that the phenotypic distinction associated with sarcoidosis is primarily driven by the axis of ventilatory impairment in the multivariate space. Body composition strengthens this distinction, and inflammatory markers (NLR) and age provide secondary and auxiliary information. The visual outputs of the model (**Figure 4**) consistently support both the direction of the distinction and the relative order of the contribution of the variables.

### 3.6 Disease stage-based comparisons

**Figure 5:**
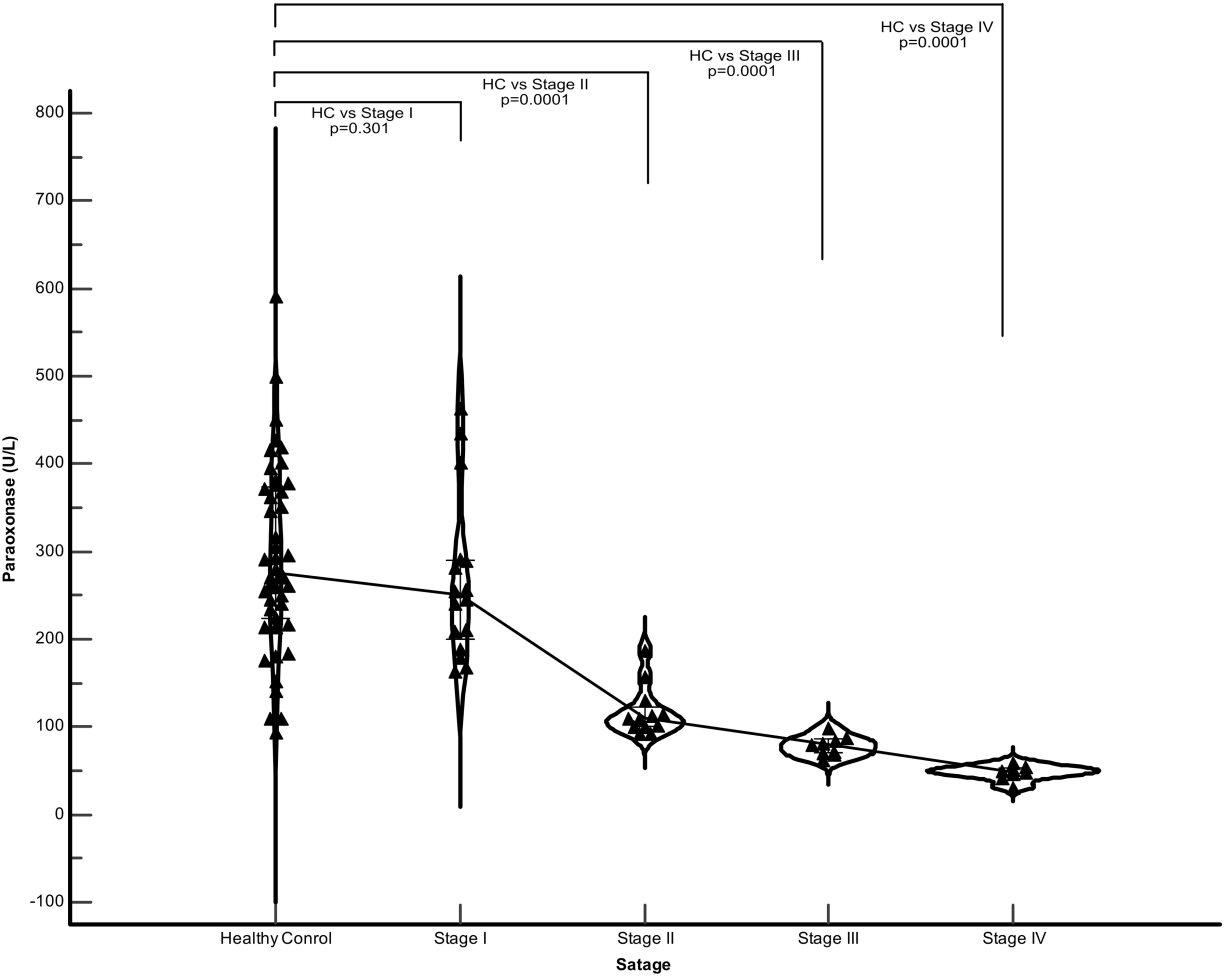
Distribution of serum paraoxonase levels according to the clinical stages of sarcoidosis (Stage I–IV). The violin plots illustrate the density and spread of PON1 activity across the four stages, with central lines indicating medians and interquartile ranges. A progressive and statistically significant decline in serum PON1 activity was observed as the disease stage advanced (Stage I > Stage II > Stage III > Stage IV), reflecting an inverse relationship between antioxidant status and disease severity.

The demographic, clinical, and laboratory characteristics of the participants included in the study were grouped according to disease stage, and their comparisons are summarized in **Table 3**. No statistically significant differences were found in age, height, weight, or BMI across disease stages. Significant changes were observed in the systemic inflammatory markers with increasing disease severity. The total WBC count was significantly higher in the Stage IV group than in the other groups. Similarly, the neutrophil count showed a gradual and significant increase from stage I to IV. While no significant change was observed in lymphocyte counts between the stages (p>0.05), this was reflected in the NLR. NLR values ​​were significantly higher in Stage II than in Stage I and in Stages III and IV than in Stages I and II (p<0.05). Platelet count was also found to be significantly higher in Stage II than in Stage I, and in Stages III and IV than in the first two stages. All pulmonary function parameters showed statistically significant deterioration with increasing disease stage (**Table 3**). FEV1, FEV1%, and FVC values ​​were significantly lower in stage III and IV patients than in stage I and II patients. Similarly, (FVC%) showed a significant decrease in Stage II compared to Stage I, and in Stages III and IV compared to the first two stages. The DLCO value, which shows gas exchange capacity, was significantly lower in Stage IV than in all other groups and showed a gradual decrease with disease severity. When sarcoidosis subgroups were compared individually with healthy controls, Stage I patients did not show a statistically significant reduction in PON1 activity (p=0.301), although median values were numerically lower. A statistically significant decline in PON1 activity became evident starting from Stage II (p<0.001) compared to controls, and this significant reduction persisted in Stage III (p<0.001) and Stage IV (p<0.001) (**Figure 5**). Paraoxonase levels in Stages II, III and IV were significantly lower than those in Stage I (p<0.05). In contrast, no statistically significant change was observed in serum glucose, urea, creatinine, ALT, and AST levels among the four stages (p>0.05).

**Table 3:** Comparative analysis of demographic, anthropometric, hematological, biochemical, and respiratory function parameters in stage I, II, III and IV.

		Stage I (N = 16)	Stage II (N = 12)	Stage III (N = 9)	Stage IV (N = 8)
Gender	Female	5	9	7	4
	Male	11	3	2	4
Age (year)		48.58 ± 23.25^a^	55.7 ± 10.2^a^	53.1 ± 11.1^a^	49.2 ± 17.1^a^
Height		174 (166-176.5)^a^	161 (154-168)^a^	158 (153-171)^a^	166.5 (159-175)^a^
Weight		82 (72-89.5)^a^	74 (72-82.5)^a^	82 (80-85)^a^	74.5 (66-84)^a^
BMI (kg/m2)		26.5 (25.7-28.4)^a^	27.2 (26.3-32.2)^a^	27.3 (24.4-35.1)^a^	26.5 (24.7-28.1)^a^
WBC (10^3^/μL)		8.4 ± 0.5^a^	9.7 ± 0.7^b^	10.5 ± 0.6^b,c^	12.7 ± 0.5^c,d^
RBC (10^6^/μL)		4.64 (4.61-4.75)^a^	4.60 (4.56-4.64)^a^	4.52 (4.5-4.53)^b^	4.39 (4.37-4.41)^b,c^
Neutrophil (10^3^/μL)		4.1 ± 1^a^	6.1 ± 0.4^b^	7.2 ± 0.4^b,c^	9 ± 0.7^c^
Lymphocyte (10^3^/μL)		2.3 (1.9-2.4)^a^	2.5 (2.4-2.6)^a^	2.5 (2.4-2.5)^a^	3 (2.9-3.1)^a^
Platelet (10^3^/μL)		283 (274-307)^a^	329(324-339)^b^	376 (353-382)^c^	401 (392.5-423.5)^c^
NLR		1.73 (1.57-2.06)^a^	2.47 (2.31-2.55)^b^	2.91 (2.72-3.08)^b,c^	3.01 (2.834-3.23)^c^
Glucose (mg/dL)		87.7 (81.65-91.8)^a^	92.9 (85.8-97)^a^	83.5 (81.2-95)^a^	86.15 (81.2-102)^a^
Urea (mg/dL)		30.6 ± 4.64^a^	27.8 ± 4.86^a^	31.2 ± 7.71^a^	31.7 ± 2.99^a^
Creatinine (mg/dL)		0.95 (0.87-1.045)^a^	0.92 (0.85-0.98)^a^	0.95 (0.93-1.06)^a^	0.86 (0.81-0.99)^a^
ALT (U/L)		22.9 (19.4-29.4)^a^	29.3 (24.1-52.6)^a^	25.4 (24.9-31.5)^a^	23.4 (21.15-23.7)^a^
AST (U/L)		23.6 (17.85-28.1)^a^	22.5 (18.7-40.7)^a^	21.8 (19.4-25)^a^	23.8 (20.15-30.65)^a^
FEV1		2.7 (2.55-2.95)^a^	2.35 (2.1-2.5)^a^	1.1 (1-1.3)^b^	0.8 (0.55-0.9)^b^
FEV1%		85.3 (83.4-86.4)^a^	75.15 (73.1-77)^a^	64.8 (63.4-66)^b^	57 (53.8-57.8)^b^
FVC L		3.44 (3.12-3.62)^a^	3.12 (2.9-3.24)^a^	1.6 (1.4-1.8)^b^	1.1 (0.95-1.33)^b^
FVC %		91.7 (89.4-93.4)^a^	81.35 (79.4-83.3)^b^	73.9 (70.7-75.4)^c^	64.4 (62.35-65.3)^c^
FEV1/FVC		80.6 (77.6-85.44)^a^	77.5 (72.1-80.4)^a,b^	71.44 (66.7-75)^a,b^	66.1 (58.66-78.6)^b^
DLCO		83.25 (79.3-85.1)^a^	71.2 (69.4-76.7)^a,b^	65 (64.1-66.3)^b,c^	57.3 (55.1-59.9)^c^
Paraoxonase (U/L)		249 (199-290)^a^	110 (100-122)^b,c^	79.8 (70.9-85.28)^c^	49.2 (43.9-52.7)^c,d^

Different letters in the same row indicated a statistically significant difference at the p<0.05 level. ALT, Alanine aminotransferase; AST, Aspartate aminotransferase; BMI, Body mass index; DLCO, Diffusing capacity of the lungs for carbon monoxide; FEV1, Forced expiratory volume in one second; FVC, Forced vital capacity; NLR, Neutrophil-to-lymphocyte ratio; Paraoxonase, Paraoxonase enzyme activity; RBC, Red blood cell; WBC, White blood cell.

In the correlation analysis between paraoxanase and age, BMI, FEV1, FVC, and DLCO values ​​in the sarcoidosis patient group, a statistically significant negative correlation was found between NLR and FEV1, FVC, DLCO, and paraoxanase values ​​(r=-0.779, r=-0.747, r=-0.718, and r=-0.830, respectively) (**Supplementary Figure S1-Supplementary File**). Furthermore, statistically significant positive correlations were found between paraoxanase-FEV1 (r=0.835), paraoxanase-FVC (r=0.807), and paraoxanase-DLCO (r=0.905) (**Supplementary Figure S1-Supplementary File**).

## 4 Discussion

In this study, serum PON1 activity was found to be significantly lower in patients with sarcoidosis than in healthy controls. This result suggests that oxidative stress is increased and the antioxidant defense capacity is reduced in sarcoidosis. PON1 is an enzyme associated with high-density lipoprotein (HDL) that protects cells from oxidative damage by hydrolyzing lipid peroxides. The observed low PON1 activity may be related to the increased oxidative burden caused by reactive oxygen species (ROS) released from activated macrophages during granulomatous inflammation. This finding is consistent with existing literature suggesting that oxidative stress plays an important role in the pathogenesis of sarcoidosis (7, 23).

It is worth noting that a broad variability in serum PON1 activity was observed within the healthy control group, with some values falling below 200 U/L. This distribution likely reflects the physiological influence of PON1 genetic polymorphisms (such as Q192R), which represent a well-known source of inter-individual variability in enzyme activity independent of disease status.

In our study, a significant positive correlation was observed between PON1 levels and lung function parameters (FEV_1_, FVC, and DLCO). This relationship demonstrates that antioxidant activity is important for maintaining lung function. The ability of PON1 to neutralize lipid peroxides may support gas exchange efficiency by maintaining alveolar epithelial integrity. These results suggest that PON1 may play an important role in maintaining redox balance not only in the systemic circulation but also at the lung level. Increased oxidative stress in sarcoidosis is known to be associated with clinical outcomes such as alveolar epithelial damage and fibrosis, PON1 deficiency may facilitate the pulmonary effects of sarcoidosis. Consistent with our stage analysis, Uzun et al. reported significantly lower PON1 levels in active and inactive sarcoidosis (22). Taken together, these observations demonstrate that PON1 levels decrease with increasing disease severity, highlighting its potential as an activity marker for sarcoidosis. Future studies incorporating direct lipid peroxidation biomarkers, including malondialdehyde (MDA), 8-iso-PGF2α, 4-hydroxynonenal (4-HNE), and lipid hydroperoxides (LOOH), are required to validate and expand our findings. In addition, paraoxonase activity measurements should be complemented with arylesterase assays to provide a more comprehensive assessment of the overall PON1 function.

ROC curve analysis showed that PON1 could distinguish sarcoidosis patients from controls, with an AUC of 0.838. An AUC of 0.838 indicated fairly good discriminatory ability. Using a cut-off of ≤210.53 U/L, we achieved 75% sensitivity and 82% specificity. These performance metrics imply that although PON1 alone is not a definitive diagnostic test, it is a promising biomarker for sarcoidosis after further validation. In general, AUC quantifies how well a test separates diseased individuals from healthy individuals, and our results suggest that PON1 has considerable predictive accuracy in this context. However, the diagnostic threshold identified in our study requires validation in independent cohorts before clinical implementation.

Although paraoxonase activity was significantly associated with sarcoidosis in the unadjusted analyses, this relationship did not remain independent in the multivariate logistic regression model. After adjusting for potential confounders, such as lung function parameters and BMI, the predictive value of PON1 was attenuated. This suggests that the apparent protective effect of PON1 may be partially mediated by its correlation with other clinical characteristics rather than representing a standalone predictor. Therefore, PON1 activity may be more reflective of overall disease severity and oxidative burden than an independent diagnostic indicator. Assay sensitivity was meticulously monitored to minimize the risk of falsely underestimating biomarkers, which is a known concern. Stable calibration performance, low intra-assay variability, and the use of freshly prepared substrates support the accuracy of our PON1 activity measurements.

The biochemical basis linking PON1 to oxidative stress provides important mechanistic context for our findings. PON1 is an HDL-associated enzyme with potent antioxidant properties, primarily through its ability to hydrolyze oxidized phospholipids, lipid peroxides, and lactone-containing substrates that are generated during oxidative injury. By preventing the accumulation of oxidized lipids and preserving HDL’s anti-inflammatory capacity of HDL, PON1 plays a critical role in modulating redox homeostasis. Under conditions of heightened oxidative stress, such as chronic granulomatous inflammation in sarcoidosis, reactive oxygen species can directly impair PON1 structure, reduce its catalytic efficiency, or alter HDL composition in a manner that reduces enzyme stability. Consequently, systemic oxidative burden may decrease circulating PON1 activity and amplify lipid peroxidation, creating a self-reinforcing cycle that contributes to the pathophysiology of sarcoidosis.

In the multivariate PLS-DA model, the variables that most strongly distinguished sarcoidosis patients from controls were FEV1, FVC, and body mass index (BMI). This underscores the importance of pulmonary function and body composition in patients with sarcoidosis. Notably, Tunç et al. (24) recently reported that sarcoidosis patients had significantly higher BMI than matched controls, consistent with our observations. Elevated BMI and adiposity may contribute to systemic inflammation and mechanically affect lung function. Thus, these data suggest that both respiratory impairment and increased body fat characterized the sarcoidosis group in our cohort.

Consistent with PLS-DA findings, multivariable logistic regression showed that reduced FEV1 and higher BMI were independently associated with sarcoidosis, whereas PON1 was not significant when these covariates were included. This indicates that lung impairment and adiposity are primary predictors of disease status, potentially overshadowing the direct effects of PON1. The strong link between a higher BMI and sarcoidosis is consistent with the idea that adipose-related inflammation can exacerbate granulomatous disease. Conversely, preserved lung function appears to be protective or indicative of a mild disease. Therefore, while PON1 was associated with sarcoidosis in univariate analysis, its predictive power diminished after accounting for pulmonary function and metabolic factors.

A notable finding of our study is that PON1 activity in Stage I patients remained comparable to healthy controls, contrasting with the sharp decline observed in Stage II. This suggests that in the early phase of sarcoidosis, characterized primarily by hilar lymphadenopathy, the systemic oxidative burden is either minimal or well-compensated. The significant depletion of PON1 starting from Stage II indicates that oxidative stress becomes a dominant feature with the onset of parenchymal infiltration and granulomatous burden. Therefore, reduced antioxidant capacity appears to be a consequence of disease progression and tissue damage rather than an initiating factor present throughout the entire disease course. Previous studies have also reported decreased PON1 activity in patients with active or advanced sarcoidosis (21, 22). Therefore, PON1 can be considered as a potential biomarker that varies in parallel with disease activity and stage.

Reduced PON1 activity has been described in a wide range of inflammatory and oxidative-stress–related conditions, including atherosclerotic cardiovascular disease, diabetes mellitus, chronic obstructive pulmonary disease, rheumatoid arthritis, and other autoimmune disorders. These observations suggest that diminished PON1 activity may be a more generalizable marker of systemic oxidative burden than a disease-specific phenomenon. Accordingly, the reduction in PON1 observed in our sarcoidosis cohort likely reflects heightened oxidative stress within the granulomatous inflammatory environment but is not unique to sarcoidosis. This broader context supports the interpretation of PON1 as a complementary indicator of oxidative imbalance, rather than a sarcoidosis-specific biomarker.

A conceptual limitation of the present study is the reliance on a single biochemical marker to characterize or discriminate complex, idiopathic, and heterogeneous diseases such as sarcoidosis. Given the wide spectrum of inflammatory, immunological, and oxidative phenotypes observed in sarcoidosis, it is unlikely that a single biomarker would respond uniformly across all patient subgroups. In this context, PON1 activity may be more appropriately interpreted as a complementary indicator that reflects one dimension of the oxidative and metabolic milieu, rather than as a standalone diagnostic tool. Therefore, the integration of PON1 with additional inflammatory, metabolic, or oxidative stress biomarkers may provide a more comprehensive assessment of disease biology in future studies.

### 4.1 Limitations

This study had several limitations. First, our sample size was limited and focused on a single-center cohort; therefore, the generalizability of our results may be limited. Second, genetic polymorphisms, liver function, nutritional status, and certain medications that may affect PON1 levels were not controlled in this study. Third, owing to the cross-sectional design of our study, causality between changes in PON1 levels and disease progression cannot be definitively established. Furthermore, other biomarkers (e.g., malondialdehyde and total antioxidant capacity) were not measured to provide a more comprehensive assessment of oxidative stress. Another important limitation of this study is the absence of widely accepted and well-validated oxidative stress biomarkers, such as MDA, 8-iso-PGF2α, 4-HNE, and LOOH. The inclusion of these markers could have provided stronger biochemical corroboration for oxidative damage and allowed a more direct evaluation of the relationship between PON1 activity and oxidative stress. Therefore, our interpretation primarily relies on indirect inferences. In addition, the measurement of PON1 activity relied solely on paraoxon-based colorimetric assays. As arylesterase activity, an essential complementary indicator of total PON1 catalytic capacity, was not assessed, our evaluation reflects only paraoxonase-specific activity rather than global PON1 function. Another methodological consideration is the absence of PON1 genotyping (e.g., Q192R and L55M polymorphisms), which are known to cause substantial interindividual variability in enzyme activity. Because this study was conducted in a single-center Turkish cohort, the population-specific genetic background may have influenced the baseline PON1 activity levels and affected the interpretation of group comparisons. In addition, the proposed diagnostic cutoff for PON1 has not been validated in an external cohort. Given that PON1 levels may vary according to genetic background, assay conditions, and disease severity, external validation is essential to confirm the generalizability and clinical utility of this threshold. These limitations necessitate caution when interpreting the findings, and future studies should be multicenter, longitudinal, and employ larger biomarker panels. Finally, although PLS-DA provided a robust visualization of group separation, we acknowledge that its application to a low-dimensional clinical dataset carries a risk of overfitting; therefore, these multivariate results should be interpreted as exploratory and complementary to the logistic regression analysis. Despite these limitations, our findings provide preliminary but meaningful insights into the potential role of PON1 in oxidative imbalance.

## 5 Conclusion

This study demonstrated that serum PON1 activity is significantly lower in sarcoidosis patients than in healthy controls. A gradual decrease in PON1 levels was observed as the disease progressed, suggesting increased oxidative stress and decreased antioxidant defense. The positive correlation between PON1 levels and lung function parameters supports the potential of this enzyme to reflect disease activity. ROC analysis demonstrated that PON1 provides significant accuracy in distinguishing patients with sarcoidosis from healthy individuals. However, multivariate analyses revealed that factors, such as lung function and BMI, limited the predictive power of PON1. These findings suggest that while PON1 activity is preserved in Stage I, it serves as a significant marker of oxidative progression starting from Stage II. Therefore, PON1 is valuable for monitoring disease severity and chronicity rather than early-stage diagnosis.

## Data Availability

The original contributions presented in the study are included in the article/**Supplementary Material**. Further inquiries can be directed to the corresponding authors.
