# Supplementary material for: Serum paraoxonase-1 as a marker of oxidative stress and pulmonary dysfunction in sarcoidosis: association with disease activity and prognostic potential
Source: Front Immunol. 2026 Jan 12;16:1731991. doi: 10.3389/fimmu.2025.1731991 (PMC12832362; doi:10.3389/fimmu.2025.1731991)
Supplement: Supplementary file 1 [file DataSheet1.docx]

**Table S1.** The All-Correlations

|  | | Age (year) | Height | Weight | BMI (kg/m2) | | WBC (103/μL) | | RBC (106/μL) | | Neutrophil (103/μL) | | Lymphocyte (103/μL) | | Platelet (103/μL) | | NLR | | Glucose (mg/dL) | | Urea (mg/dL) | Creatinine (mg/dL) | | ALT (U/L) | AST (U/L) | | FEV1 | | FEV1 % | | FVC litre | | FVC % | | FEV1 / FVC | | DLCO | | Paraoxonase (U/L) | |
| --- | --- | --- | --- | --- | --- | --- | --- | --- | --- | --- | --- | --- | --- | --- | --- | --- | --- | --- | --- | --- | --- | --- | --- | --- | --- | --- | --- | --- | --- | --- | --- | --- | --- | --- | --- | --- | --- | --- | --- | --- |
| Spearman's rho | Age (year) | Correlation Coefficient | | 1,000 | -,074 | ,100 | | ,248^*^ | | ,226^*^ | | -,074 | | ,028 | | ,071 | | ,027 | | -,059 | ,087 | -,145 | ,116 | | ,081 | -,058 | | -,223^*^ | | -,130 | | -,241^*^ | | -,057 | | -,136 | | -,167 | | -,053 |
|  |  | Sig. (2-tailed) | | . | ,488 | ,350 | | ,018 | | ,032 | | ,489 | | ,795 | | ,508 | | ,802 | | ,581 | ,415 | ,173 | ,277 | | ,446 | ,589 | | ,035 | | ,222 | | ,022 | | ,591 | | ,202 | | ,116 | | ,617 |
|  |  | N | | 90 | 90 | 90 | | 90 | | 90 | | 90 | | 90 | | 90 | | 90 | | 90 | 90 | 90 | 90 | | 90 | 90 | | 90 | | 90 | | 90 | | 90 | | 90 | | 90 | | 90 |
|  | Height | Correlation Coefficient | | -,074 | 1,000 | ,654^**^ | | -,123 | | -,336^**^ | | ,232^*^ | | -,212^*^ | | -,130 | | -,151 | | -,156 | -,039 | ,001 | -,178 | | ,004 | ,165 | | ,207^*^ | | ,210^*^ | | ,244^*^ | | ,205 | | ,216^*^ | | ,228^*^ | | ,216^*^ |
|  |  | Sig. (2-tailed) | | ,488 | . | ,000 | | ,246 | | ,001 | | ,028 | | ,045 | | ,224 | | ,155 | | ,141 | ,715 | ,990 | ,094 | | ,967 | ,121 | | ,050 | | ,047 | | ,020 | | ,053 | | ,041 | | ,031 | | ,041 |
|  |  | N | | 90 | 90 | 90 | | 90 | | 90 | | 90 | | 90 | | 90 | | 90 | | 90 | 90 | 90 | 90 | | 90 | 90 | | 90 | | 90 | | 90 | | 90 | | 90 | | 90 | | 90 |
|  | Weight | Correlation Coefficient | | ,100 | ,654^**^ | 1,000 | | ,517^**^ | | -,045 | | ,119 | | ,011 | | ,079 | | ,039 | | -,087 | -,066 | -,194 | -,113 | | -,005 | ,015 | | -,107 | | -,087 | | -,009 | | -,006 | | -,174 | | -,067 | | -,008 |
|  |  | Sig. (2-tailed) | | ,350 | ,000 | . | | ,000 | | ,676 | | ,264 | | ,915 | | ,458 | | ,714 | | ,414 | ,537 | ,067 | ,287 | | ,964 | ,886 | | ,315 | | ,415 | | ,936 | | ,958 | | ,101 | | ,531 | | ,944 |
|  |  | N | | 90 | 90 | 90 | | 90 | | 90 | | 90 | | 90 | | 90 | | 90 | | 90 | 90 | 90 | 90 | | 90 | 90 | | 90 | | 90 | | 90 | | 90 | | 90 | | 90 | | 90 |
|  | BMI (kg/m2) | Correlation Coefficient | | ,248^*^ | -,123 | ,517^**^ | | 1,000 | | ,245^*^ | | -,139 | | ,145 | | ,212^*^ | | ,206 | | -,012 | -,008 | -,278^**^ | -,002 | | -,004 | -,112 | | -,301^**^ | | -,262^*^ | | -,206 | | -,118 | | -,417^**^ | | -,333^**^ | | -,210^*^ |
|  |  | Sig. (2-tailed) | | ,018 | ,246 | ,000 | | . | | ,020 | | ,193 | | ,172 | | ,045 | | ,052 | | ,910 | ,942 | ,008 | ,983 | | ,971 | ,294 | | ,004 | | ,013 | | ,051 | | ,270 | | ,000 | | ,001 | | ,047 |
|  |  | N | | 90 | 90 | 90 | | 90 | | 90 | | 90 | | 90 | | 90 | | 90 | | 90 | 90 | 90 | 90 | | 90 | 90 | | 90 | | 90 | | 90 | | 90 | | 90 | | 90 | | 90 |
|  | WBC (103/μL) | Correlation Coefficient | | ,226^*^ | -,336^**^ | -,045 | | ,245^*^ | | 1,000 | | -,340^**^ | | ,425^**^ | | ,445^**^ | | ,351^**^ | | ,114 | ,092 | -,013 | ,076 | | ,074 | -,226^*^ | | -,673^**^ | | -,700^**^ | | -,673^**^ | | -,565^**^ | | -,491^**^ | | -,708^**^ | | -,508^**^ |
|  |  | Sig. (2-tailed) | | ,032 | ,001 | ,676 | | ,020 | | . | | ,001 | | ,000 | | ,000 | | ,001 | | ,286 | ,390 | ,903 | ,477 | | ,490 | ,032 | | ,000 | | ,000 | | ,000 | | ,000 | | ,000 | | ,000 | | ,000 |
|  |  | N | | 90 | 90 | 90 | | 90 | | 90 | | 90 | | 90 | | 90 | | 90 | | 90 | 90 | 90 | 90 | | 90 | 90 | | 90 | | 90 | | 90 | | 90 | | 90 | | 90 | | 90 |
|  | RBC (106/μL) | Correlation Coefficient | | -,074 | ,232^*^ | ,119 | | -,139 | | -,340^**^ | | 1,000 | | -,210^*^ | | -,165 | | -,325^**^ | | -,162 | ,141 | -,051 | ,167 | | ,110 | -,031 | | ,188 | | ,255^*^ | | ,227^*^ | | ,299^**^ | | ,156 | | ,326^**^ | | ,278^**^ |
|  |  | Sig. (2-tailed) | | ,489 | ,028 | ,264 | | ,193 | | ,001 | | . | | ,047 | | ,120 | | ,002 | | ,126 | ,184 | ,635 | ,116 | | ,301 | ,770 | | ,076 | | ,015 | | ,032 | | ,004 | | ,143 | | ,002 | | ,008 |
|  |  | N | | 90 | 90 | 90 | | 90 | | 90 | | 90 | | 90 | | 90 | | 90 | | 90 | 90 | 90 | 90 | | 90 | 90 | | 90 | | 90 | | 90 | | 90 | | 90 | | 90 | | 90 |
|  | Neutrophil (103/μL) | Correlation Coefficient | | ,028 | -,212^*^ | ,011 | | ,145 | | ,425^**^ | | -,210^*^ | | 1,000 | | ,446^**^ | | ,596^**^ | | ,631^**^ | -,115 | -,016 | ,087 | | ,087 | -,003 | | -,430^**^ | | -,566^**^ | | -,461^**^ | | -,652^**^ | | -,269^*^ | | -,476^**^ | | -,566^**^ |
|  |  | Sig. (2-tailed) | | ,795 | ,045 | ,915 | | ,172 | | ,000 | | ,047 | | . | | ,000 | | ,000 | | ,000 | ,278 | ,882 | ,413 | | ,414 | ,979 | | ,000 | | ,000 | | ,000 | | ,000 | | ,010 | | ,000 | | ,000 |
|  |  | N | | 90 | 90 | 90 | | 90 | | 90 | | 90 | | 90 | | 90 | | 90 | | 90 | 90 | 90 | 90 | | 90 | 90 | | 90 | | 90 | | 90 | | 90 | | 90 | | 90 | | 90 |
|  | Lymphocyte (103/μL) | Correlation Coefficient | | ,071 | -,130 | ,079 | | ,212^*^ | | ,445^**^ | | -,165 | | ,446^**^ | | 1,000 | | ,427^**^ | | -,299^**^ | ,016 | ,121 | ,110 | | -,052 | -,039 | | -,481^**^ | | -,504^**^ | | -,494^**^ | | -,422^**^ | | -,258^*^ | | -,481^**^ | | -,521^**^ |
|  |  | Sig. (2-tailed) | | ,508 | ,224 | ,458 | | ,045 | | ,000 | | ,120 | | ,000 | | . | | ,000 | | ,004 | ,881 | ,257 | ,301 | | ,625 | ,716 | | ,000 | | ,000 | | ,000 | | ,000 | | ,014 | | ,000 | | ,000 |
|  |  | N | | 90 | 90 | 90 | | 90 | | 90 | | 90 | | 90 | | 90 | | 90 | | 90 | 90 | 90 | 90 | | 90 | 90 | | 90 | | 90 | | 90 | | 90 | | 90 | | 90 | | 90 |
|  | Platelet (103/μL) | Correlation Coefficient | | ,027 | -,151 | ,039 | | ,206 | | ,351^**^ | | -,325^**^ | | ,596^**^ | | ,427^**^ | | 1,000 | | ,233^*^ | ,117 | ,073 | ,125 | | -,058 | -,048 | | -,432^**^ | | -,546^**^ | | -,437^**^ | | -,470^**^ | | -,262^*^ | | -,526^**^ | | -,577^**^ |
|  |  | Sig. (2-tailed) | | ,802 | ,155 | ,714 | | ,052 | | ,001 | | ,002 | | ,000 | | ,000 | | . | | ,027 | ,274 | ,495 | ,240 | | ,584 | ,656 | | ,000 | | ,000 | | ,000 | | ,000 | | ,013 | | ,000 | | ,000 |
|  |  | N | | 90 | 90 | 90 | | 90 | | 90 | | 90 | | 90 | | 90 | | 90 | | 90 | 90 | 90 | 90 | | 90 | 90 | | 90 | | 90 | | 90 | | 90 | | 90 | | 90 | | 90 |
|  | NLR | Correlation Coefficient | | -,059 | -,156 | -,087 | | -,012 | | ,114 | | -,162 | | ,631^**^ | | -,299^**^ | | ,233^*^ | | 1,000 | -,142 | -,139 | -,051 | | ,040 | -,015 | | -,021 | | -,158 | | -,065 | | -,306^**^ | | -,038 | | -,119 | | -,147 |
|  |  | Sig. (2-tailed) | | ,581 | ,141 | ,414 | | ,910 | | ,286 | | ,126 | | ,000 | | ,004 | | ,027 | | . | ,183 | ,191 | ,631 | | ,708 | ,889 | | ,847 | | ,138 | | ,546 | | ,003 | | ,721 | | ,265 | | ,166 |
|  |  | N | | 90 | 90 | 90 | | 90 | | 90 | | 90 | | 90 | | 90 | | 90 | | 90 | 90 | 90 | 90 | | 90 | 90 | | 90 | | 90 | | 90 | | 90 | | 90 | | 90 | | 90 |
|  | Glucose (mg/dL) | Correlation Coefficient | | ,087 | -,039 | -,066 | | -,008 | | ,092 | | ,141 | | -,115 | | ,016 | | ,117 | | -,142 | 1,000 | -,069 | ,026 | | ,123 | -,005 | | -,102 | | -,037 | | -,099 | | -,022 | | -,007 | | ,044 | | -,059 |
|  |  | Sig. (2-tailed) | | ,415 | ,715 | ,537 | | ,942 | | ,390 | | ,184 | | ,278 | | ,881 | | ,274 | | ,183 | . | ,517 | ,804 | | ,250 | ,963 | | ,339 | | ,729 | | ,355 | | ,839 | | ,949 | | ,680 | | ,581 |
|  |  | N | | 90 | 90 | 90 | | 90 | | 90 | | 90 | | 90 | | 90 | | 90 | | 90 | 90 | 90 | 90 | | 90 | 90 | | 90 | | 90 | | 90 | | 90 | | 90 | | 90 | | 90 |
|  | Urea (mg/dL) | Correlation Coefficient | | -,145 | ,001 | -,194 | | -,278^**^ | | -,013 | | -,051 | | -,016 | | ,121 | | ,073 | | -,139 | -,069 | 1,000 | ,128 | | -,204 | ,044 | | ,081 | | ,142 | | ,010 | | -,041 | | ,201 | | ,100 | | ,055 |
|  |  | Sig. (2-tailed) | | ,173 | ,990 | ,067 | | ,008 | | ,903 | | ,635 | | ,882 | | ,257 | | ,495 | | ,191 | ,517 | . | ,229 | | ,054 | ,678 | | ,446 | | ,180 | | ,926 | | ,702 | | ,057 | | ,350 | | ,604 |
|  |  | N | | 90 | 90 | 90 | | 90 | | 90 | | 90 | | 90 | | 90 | | 90 | | 90 | 90 | 90 | 90 | | 90 | 90 | | 90 | | 90 | | 90 | | 90 | | 90 | | 90 | | 90 |
|  | Creatinine (mg/dL) | Correlation Coefficient | | ,116 | -,178 | -,113 | | -,002 | | ,076 | | ,167 | | ,087 | | ,110 | | ,125 | | -,051 | ,026 | ,128 | 1,000 | | ,225^*^ | ,041 | | -,183 | | -,270^*^ | | -,150 | | -,149 | | -,163 | | -,194 | | -,127 |
|  |  | Sig. (2-tailed) | | ,277 | ,094 | ,287 | | ,983 | | ,477 | | ,116 | | ,413 | | ,301 | | ,240 | | ,631 | ,804 | ,229 | . | | ,033 | ,702 | | ,085 | | ,010 | | ,157 | | ,162 | | ,124 | | ,067 | | ,233 |
|  |  | N | | 90 | 90 | 90 | | 90 | | 90 | | 90 | | 90 | | 90 | | 90 | | 90 | 90 | 90 | 90 | | 90 | 90 | | 90 | | 90 | | 90 | | 90 | | 90 | | 90 | | 90 |
|  | ALT (U/L) | Correlation Coefficient | | ,081 | ,004 | -,005 | | -,004 | | ,074 | | ,110 | | ,087 | | -,052 | | -,058 | | ,040 | ,123 | -,204 | ,225^*^ | | 1,000 | ,211^*^ | | -,234^*^ | | -,263^*^ | | -,199 | | -,145 | | -,209^*^ | | -,035 | | -,168 |
|  |  | Sig. (2-tailed) | | ,446 | ,967 | ,964 | | ,971 | | ,490 | | ,301 | | ,414 | | ,625 | | ,584 | | ,708 | ,250 | ,054 | ,033 | | . | ,046 | | ,026 | | ,012 | | ,061 | | ,173 | | ,048 | | ,744 | | ,115 |
|  |  | N | | 90 | 90 | 90 | | 90 | | 90 | | 90 | | 90 | | 90 | | 90 | | 90 | 90 | 90 | 90 | | 90 | 90 | | 90 | | 90 | | 90 | | 90 | | 90 | | 90 | | 90 |
|  | AST (U/L) | Correlation Coefficient | | -,058 | ,165 | ,015 | | -,112 | | -,226^*^ | | -,031 | | -,003 | | -,039 | | -,048 | | -,015 | -,005 | ,044 | ,041 | | ,211^*^ | 1,000 | | ,077 | | ,064 | | ,120 | | -,026 | | -,047 | | ,085 | | -,008 |
|  |  | Sig. (2-tailed) | | ,589 | ,121 | ,886 | | ,294 | | ,032 | | ,770 | | ,979 | | ,716 | | ,656 | | ,889 | ,963 | ,678 | ,702 | | ,046 | . | | ,472 | | ,547 | | ,260 | | ,809 | | ,661 | | ,428 | | ,942 |
|  |  | N | | 90 | 90 | 90 | | 90 | | 90 | | 90 | | 90 | | 90 | | 90 | | 90 | 90 | 90 | 90 | | 90 | 90 | | 90 | | 90 | | 90 | | 90 | | 90 | | 90 | | 90 |
|  | FEV1 | Correlation Coefficient | | -,223^*^ | ,207^*^ | -,107 | | -,301^**^ | | -,673^**^ | | ,188 | | -,430^**^ | | -,481^**^ | | -,432^**^ | | -,021 | -,102 | ,081 | -,183 | | -,234^*^ | ,077 | | 1,000 | | ,687^**^ | | ,925^**^ | | ,534^**^ | | ,712^**^ | | ,761^**^ | | ,722^**^ |
|  |  | Sig. (2-tailed) | | ,035 | ,050 | ,315 | | ,004 | | ,000 | | ,076 | | ,000 | | ,000 | | ,000 | | ,847 | ,339 | ,446 | ,085 | | ,026 | ,472 | | . | | ,000 | | ,000 | | ,000 | | ,000 | | ,000 | | ,000 |
|  |  | N | | 90 | 90 | 90 | | 90 | | 90 | | 90 | | 90 | | 90 | | 90 | | 90 | 90 | 90 | 90 | | 90 | 90 | | 90 | | 90 | | 90 | | 90 | | 90 | | 90 | | 90 |
|  | FEV1 % | Correlation Coefficient | | -,130 | ,210^*^ | -,087 | | -,262^*^ | | -,700^**^ | | ,255^*^ | | -,566^**^ | | -,504^**^ | | -,546^**^ | | -,158 | -,037 | ,142 | -,270^*^ | | -,263^*^ | ,064 | | ,687^**^ | | 1,000 | | ,595^**^ | | ,579^**^ | | ,638^**^ | | ,776^**^ | | ,673^**^ |
|  |  | Sig. (2-tailed) | | ,222 | ,047 | ,415 | | ,013 | | ,000 | | ,015 | | ,000 | | ,000 | | ,000 | | ,138 | ,729 | ,180 | ,010 | | ,012 | ,547 | | ,000 | | . | | ,000 | | ,000 | | ,000 | | ,000 | | ,000 |
|  |  | N | | 90 | 90 | 90 | | 90 | | 90 | | 90 | | 90 | | 90 | | 90 | | 90 | 90 | 90 | 90 | | 90 | 90 | | 90 | | 90 | | 90 | | 90 | | 90 | | 90 | | 90 |
|  | FVC litre | Correlation Coefficient | | -,241^*^ | ,244^*^ | -,009 | | -,206 | | -,673^**^ | | ,227^*^ | | -,461^**^ | | -,494^**^ | | -,437^**^ | | -,065 | -,099 | ,010 | -,150 | | -,199 | ,120 | | ,925^**^ | | ,595^**^ | | 1,000 | | ,545^**^ | | ,441^**^ | | ,701^**^ | | ,662^**^ |
|  |  | Sig. (2-tailed) | | ,022 | ,020 | ,936 | | ,051 | | ,000 | | ,032 | | ,000 | | ,000 | | ,000 | | ,546 | ,355 | ,926 | ,157 | | ,061 | ,260 | | ,000 | | ,000 | | . | | ,000 | | ,000 | | ,000 | | ,000 |
|  |  | N | | 90 | 90 | 90 | | 90 | | 90 | | 90 | | 90 | | 90 | | 90 | | 90 | 90 | 90 | 90 | | 90 | 90 | | 90 | | 90 | | 90 | | 90 | | 90 | | 90 | | 90 |
|  | FVC % | Correlation Coefficient | | -,057 | ,205 | -,006 | | -,118 | | -,565^**^ | | ,299^**^ | | -,652^**^ | | -,422^**^ | | -,470^**^ | | -,306^**^ | -,022 | -,041 | -,149 | | -,145 | -,026 | | ,534^**^ | | ,579^**^ | | ,545^**^ | | 1,000 | | ,375^**^ | | ,512^**^ | | ,672^**^ |
|  |  | Sig. (2-tailed) | | ,591 | ,053 | ,958 | | ,270 | | ,000 | | ,004 | | ,000 | | ,000 | | ,000 | | ,003 | ,839 | ,702 | ,162 | | ,173 | ,809 | | ,000 | | ,000 | | ,000 | | . | | ,000 | | ,000 | | ,000 |
|  |  | N | | 90 | 90 | 90 | | 90 | | 90 | | 90 | | 90 | | 90 | | 90 | | 90 | 90 | 90 | 90 | | 90 | 90 | | 90 | | 90 | | 90 | | 90 | | 90 | | 90 | | 90 |
|  | FEV1 / FVC | Correlation Coefficient | | -,136 | ,216^*^ | -,174 | | -,417^**^ | | -,491^**^ | | ,156 | | -,269^*^ | | -,258^*^ | | -,262^*^ | | -,038 | -,007 | ,201 | -,163 | | -,209^*^ | -,047 | | ,712^**^ | | ,638^**^ | | ,441^**^ | | ,375^**^ | | 1,000 | | ,608^**^ | | ,543^**^ |
|  |  | Sig. (2-tailed) | | ,202 | ,041 | ,101 | | ,000 | | ,000 | | ,143 | | ,010 | | ,014 | | ,013 | | ,721 | ,949 | ,057 | ,124 | | ,048 | ,661 | | ,000 | | ,000 | | ,000 | | ,000 | | . | | ,000 | | ,000 |
|  |  | N | | 90 | 90 | 90 | | 90 | | 90 | | 90 | | 90 | | 90 | | 90 | | 90 | 90 | 90 | 90 | | 90 | 90 | | 90 | | 90 | | 90 | | 90 | | 90 | | 90 | | 90 |
|  | DLCO | Correlation Coefficient | | -,167 | ,228^*^ | -,067 | | -,333^**^ | | -,708^**^ | | ,326^**^ | | -,476^**^ | | -,481^**^ | | -,526^**^ | | -,119 | ,044 | ,100 | -,194 | | -,035 | ,085 | | ,761^**^ | | ,776^**^ | | ,701^**^ | | ,512^**^ | | ,608^**^ | | 1,000 | | ,651^**^ |
|  |  | Sig. (2-tailed) | | ,116 | ,031 | ,531 | | ,001 | | ,000 | | ,002 | | ,000 | | ,000 | | ,000 | | ,265 | ,680 | ,350 | ,067 | | ,744 | ,428 | | ,000 | | ,000 | | ,000 | | ,000 | | ,000 | | . | | ,000 |
|  |  | N | | 90 | 90 | 90 | | 90 | | 90 | | 90 | | 90 | | 90 | | 90 | | 90 | 90 | 90 | 90 | | 90 | 90 | | 90 | | 90 | | 90 | | 90 | | 90 | | 90 | | 90 |
|  | Paraoxonase (U/L) | Correlation Coefficient | | -,053 | ,216^*^ | -,008 | | -,210^*^ | | -,508^**^ | | ,278^**^ | | -,566^**^ | | -,521^**^ | | -,577^**^ | | -,147 | -,059 | ,055 | -,127 | | -,168 | -,008 | | ,722^**^ | | ,673^**^ | | ,662^**^ | | ,672^**^ | | ,543^**^ | | ,651^**^ | | 1,000 |
|  |  | Sig. (2-tailed) | | ,617 | ,041 | ,944 | | ,047 | | ,000 | | ,008 | | ,000 | | ,000 | | ,000 | | ,166 | ,581 | ,604 | ,233 | | ,115 | ,942 | | ,000 | | ,000 | | ,000 | | ,000 | | ,000 | | ,000 | | . |
|  |  | N | | 90 | 90 | 90 | | 90 | | 90 | | 90 | | 90 | | 90 | | 90 | | 90 | 90 | 90 | 90 | | 90 | 90 | | 90 | | 90 | | 90 | | 90 | | 90 | | 90 | | 90 |

**Figure S1.** Heatmap correlation matrix representing the relationships between Paraoxonase activity, age, BMI, and respiratory function parameters specifically within the sarcoidosis patient group. The color scale indicates the strength and direction of Spearman’s rank correlation coefficients; blue intensity indicates a stronger positive correlation, while red intensity indicates a stronger negative correlation.
